# We Are Not Okay

**DOI:** 10.5811/westjem.54016

**Published:** 2026-01-03

**Authors:** Deena D. Wasserman

**Affiliations:** Temple University, Department of Emergency Medicine, Philadelphia, Pennsylvania

The morning was already going to shit. I almost made it out of the house on time, but then I found ants in my granola. After I’d already sprinkled it on my carefully prepped yogurt with banana slices. I decided to sacrifice a few more minutes so I could still have half a breakfast, but I only had frozen bananas left and the second iteration was not particularly visually appealing. I ran out of my apartment with the garbage in one hand, container in the other, cursing that once again all my noble punctual intentions were bamboozled by a generous serving of executive dysfunction.

I was heading in for my least favorite shift - ED admissions. Since the hospital can’t be bothered to actually deal with their boarding issue, their solution was to create an additional shift for an attending EM physician to act as an internist and round on the boarding patients while they waited for space upstairs. It’s a thankless job consisting mostly of being yelled at by families for things completely out of your control, cajoling the nurses to not forget about the orders you put in since they still have new arrivals to deal with, and once again saying “no, I don’t know when there will be a bed on the ward” - all while trying to practice medicine outside of your specialty. They would never ask a cardiologist to consult on acute kidney injury, but somehow it’s fine for me to be adjusting blood pressure medications and evaluating hyponatremia.

I had already been informed that I was being pulled off shift for a few hours to participate in a biological mass casualty drill being run by the health ministry. When I arrived there were fifteen ED boarders, five of them over twenty four hours. I had just a couple of hours to try and see as many as I could, order home meds, request further diagnostic tests and open consults. It seems the ants were an omen. I should have just gone with my typical and chosen coffee with a side of violence.

I’m trying to calculate the appropriate basal insulin dose for the DKA-er and order antibiotics for the suspected cholangitis when the drill begins. It’s not my first time. Being disaster fellowship trained, and spending my first five years as an attending practicing during a global pandemic and multiple wars, I am used to both running and participating in all kinds of simulations. I try to ignore how many suits are here. This is why I hate the day shift.

Four bright orange bean bag dolls are unceremoniously thrown onto the gurneys in beds nine and ten. I go in with the nurse and we start the performance. As we read the information cards, the scenario becomes clear: multiple patients with high fever, some with respiratory symptoms, some with signs of meningitis. I call the infectious disease consultant and the department head to report the suspicion of a contagion. We’re told to gown up and transfer the patients to the negative pressure room. Gown, N95 mask, face shield, then gloves. I crack a joke about the perfect scenario to trigger our PTSD. The nursing supervisor gives me a dirty look and tells me to pay attention to the donning poster. We go into the room and the facilitator says that doll three, a 24-week pregnant woman, is now coding. I am joined by the nurse who pulls up the code cart, and our chief resident who half-heartedly starts tapping his palm on the doll’s middle in what are supposed to be chest compressions. I give the facilitator a wicked grin, lost behind my mask. You want to screw with me, I’m all in. I call for a thoracotomy tray and OB to get down here now for a resuscitative hysterotomy. Their faces are priceless. They decide to give me ROSC before I cut through the orange plastic and the filling spills everywhere. They tell us we can admit the patient to the ICU. I’m chuckling to myself. Meanwhile, we’re informed that incident command has declared the need to open a “biological ED” to isolate the suspected patients as they arrive. Everything is progressing as expected.

I don’t know exactly when it happened. I was fine, really. A bit hungry, since I never did eat my second attempt at breakfast; a bit annoyed that I had to participate in a fictitious disaster while the ED really was going up in flames. First, it was just the itchiness of the gown. Then, the pressure of the mask straps around my face. It was hot, and I couldn’t see because the face shield was foggy. I remember that I could never see because of the stupid face shield. And breathing, when did breathing become this hard? My chest felt tight and I heard buzzing in my ears. I looked around and there were twenty people in the room, half holding clipboards, talking and talking and talking and meanwhile I just need to leave, to get out, to take off the dumb mask and go back to my patients. But I don’t because it’s a drill and it’s not real and we have to impress the health ministry and I’m getting stank eye from nursing management as I try to adjust my mask to let in some air.

I feel it building. That panic, impending doom, the activation of my limbic system and I know exactly what is happening and still I am overwhelmed. I start with box breathing. Loud, long inhale through the nose, hold, loud, long exhale through the nose, hold. I plant my feet on the ground and lean against the wall so I can feel where every part of my body is in contact with the hard surface, willing myself to be grounded by the tactile sensation. I curl my hands into fists and dig my nails into my palms hard enough to leave a mark. I tell myself I am fine.

The resident is standing next to me chatting with the nurse, but he turns, noticing the change. He is a good friend and he knows me, recognizes that I am too quiet, too still. “You okay?” I brush it off and nod. He isn’t fooled. He keeps eyeing me.

We finally move to the biological ED. It’s our observation unit, a closed five bed ward next to the main ED, which during the COVID-19 pandemic was used to isolate the suspected and confirmed cases. The way there, the space, walking around with our gowns and masks, is familiar. The computers, the rooms, the equipment, everything is exactly as it would be in a real event, because this is exactly what we did when it really happened. And suddenly, the drill speeds up. They bring in the dolls two and three at a time. They are thrown into corners and I can’t keep track of how many patients there are and they’re supposed to be opening electronic charts for everyone, but some of them are missed. The infectious disease physician pops up once more and I ask him for an update of what to do with the patients. Antibiotics? Testing? Dispo? He gives me the recommendations, and then he leaves.

One of the facilitators comes over and starts quizzing me on appropriate doffing procedure. And I answer: yes, of course I know, first gloves and then mask and then gown, um, no, I mean gown first then mask last after cleaning my hands…. And she smiles at me condescendingly and tells me it’s ok to say I don’t remember and that I would refer to the sign that is posted in the doffing area. And I want to scream because I do remember, I was there, and I did it over and over and I never looked at any sign because I was running in between patients and who has time to look at a sign. She marks something on her clipboard and walks away, the nurse manager scurrying after her.

There are so many people walking around and looking and talking and making a fuss, but I am trying to find which patient is which and write in their charts because they’re all the same and they all have the same symptoms and everyone is alone and I have no staff and there’s no vaccine and people just keep dying and…. Wait, no. This is a drill. You are fine. This is not real. These are dolls and this already happened. But the feeling keeps growing, and I am holding on by a thread. I sit at the computer and type because it’s the only thing I can do. I don’t trust myself to open my mouth and speak or I will scream or cry or stab someone.

The nurse wants to know why I haven’t admitted anyone yet. Admit them, sure, why not. I’ll keep typing in the chart to get that going. I write the same story, the same symptoms, the same treatment for everyone. Three, four, five patients. There are still at least seven more? Eight? I’m not sure. They call me from incident command to ask for an update on how many patients. I don’t know. Ten? Twelve? The dolls are stacked in the beds and on chairs and they brought in so many in a row that not everyone has a chart yet and I’m just trying to figure out who’s who. Twelve, I say into the phone, unconfidently. And then my nurse chimes in nonchalantly, “And one deceased.”

My heart ceases to beat. I feel an impact in the center of my chest as if I just took a helmet shot from a linebacker.

“What do you mean, one deceased?”

“EMS brought one in, she’s dead.”

It’s surging again, the feeling that I’m about to tumble off the edge of a cliff. I have been clinging to the ledge for so long, watching stones crumble as I take every step, carefully, so carefully, as not to lose the footing I’ve crafted out of grit and numbness and apathy, the cheapest tender for building my fort of resilience that is based on a foundation of lies, with all the stability of a house of cards anchored to sand.

“How come I didn’t know there was one dead?” I hear the pitch of my voice rising, vibrating towards hysteria even as I fight to keep it even, steady.

The nurse has already moved on to the next doll being brought in. I get out of my chair and go to the bed in the corner room, the only one with a door instead of a curtain, where three dolls are stacked. I pretend to be reading the cards tied around their necks, gathering information, participating.

But I don’t see anything. There is a whining in my ears and the buzzing in my head is crescendoing into a roar. I feel blood rushing to my face as suddenly my heart which was stopped and frozen goes into overdrive and then all I am is the pounding of my pulse and my vision is blurred as I feel my eyes stinging. And I can’t breathe I can’t breathe I CAN’T BREATHE but I take a long inhale through my nose and I count –

*one*

because I am here and

*two*

this isn’t happening right now and

*three*

it already happened it’s over and

*four*

when I open my eyes I won’t see her, the French lady that I killed, I killed her because I didn’t know she was suffocating to death in this bed in this exact bed and I didn’t know, no one told me her blood gases weren’t normal and she was alone and her family was at home and everyone was the same and there was no space and they were here and I was here and she couldn’t breathe and I can’t breathe I can’t breathe I CAN’T CAN’T CAN’T –

*COUNT!*

*five*. Hold.

The roaring is there, but I have managed to envelope it in a translucent cage that holds it and dulls the noise. It is occupying almost all of my mental space and crowding out all logical processes and I just need to get out of here before I lose it completely. Normal human interaction is too distracting, as all my cognition is focused on keeping the tempest contained. I give myself simple tasks. Walk back to the desk. Sit down. Type. Typing I can do. The same chart, over and over. Click through the checklist. Close the admission. Open the next chart. Repeat. Breathe. Remember to breathe.

My resident, my friend sitting next to me is also in a foul mood. He is post night shift and has been in the hospital for over 24 hours. “I’m out of here, fuck this bullshit. You good?” He knows I’m not. Knows I’m not myself. But I don’t turn to him because he will see, see that my eyes have gone dead and empty and are reflecting the edge of the abyss at which I stand. I keep them on the computer, not seeing, hands on the keyboard, fingers tapping out a rhythm to keep me tethered to this time and place. I manage a single arm shrug. He claps me on the shoulder and gets up, leaving me alone amongst the nurses and the managers and the observers from the health ministry who all have no clue that I am teetering on the edge and I am barely, barely managing to keep from falling off.

My phone rings. It’s the medical director. I answer with a grunt. “We’re ending the drill, you’re dismissed.”

The pounding is back in my ears. It sounds like words now. Get out. Get out. Get out. I stand and rip off my gown. The nurse manager gives me a horrified look and ushers me to the door, to the doffing area. She starts chastising me, but I don’t really hear her. I’m dismissed, I tell her, I’m not here, I’m done, I’m done, I’m done. I pull at my N95 and the elastic snaps as I toss it in the direction of the trash, not caring to see if I missed.

Head down, I exit into the crowded ED. I need a minute, just a minute because the mask is off but I still feel the air thick and hot as I’m struggling to fill my lungs. I ignore everyone and put one foot in front of the other, not sure where I’m going, just somewhere; first to the triage room but that’s full of people, and then out to the lobby but still too many people, and I thrust my sunglasses on my face as I exit the ambulance bay doors, where I’m met by sunshine and noise and people, people everywhere. They’re chatting and smoking and drinking coffee and calling out greetings; and there’s too many of them and they can all see me and I need just a minute because I can see the monsoon coming and I don’t know how to face it because I know it won’t, but also, I am absolutely certain it will obliterate me.

Back resting against the stone pillar of the overhang, I dial my friend who was in with me. Maybe he hasn’t left yet. He answers after one ring.

“Do you have a cigarette?” I manage to get out. I don’t smoke. He knows I don’t smoke, and he quit, but sometimes on shift we stand outside and we share one while we take a break.

“No, but –“

“Okay, nevermind.” The roaring is at maximum volume.

“Don’t be stupid. One second.”

I hang up and I see a gaggle of the patient transport techs coming towards me. They stand right next to me and I know I need to move because I feel moisture on my cheeks and I can’t explain and they are going to ask me things but I can’t think, can’t move, can’t anything.

“You good, doc? You want a glass of water?”

I shake my head and contort my face into what I think is a polite smile. Apparently unconvincingly, because they start to come closer, again offering to get me something, and I back up because I just can’t –

I see my friend exit the ambulance bay doors. In three strides he is in front of me. He takes one look at my face. “Shit. We need two. Here.” He puts the cigarette between my fingers. In a word he’s bummed a second cigarette and a lighter off one of the techs. He lights his own then motions to me as he holds up the flame. I’m shaking now, so badly that my hair almost catches. I take a drag and sink onto the stone bench beside me. “No. Not here.” He grabs me by the elbow and leads me over behind the cars to the fence. People can still see us but it’s better, a little more private. He looks at me.

“What happened?”

I just stare. Take another cathartic disgusting drag from the cigarette.

“Do you want to talk about it?”

I don’t even know what to say. I open my mouth and out comes a pathetic whimper, the only thing I can express because finally, finally, finally the cresting and roaring is everywhere and the wave engulfs me as it breaks. My shoulders shake violently. I’ve forgotten how to exhale, in an effort to contain the muchness of it, and so I am silently, hysterically, sobbing, tears rolling down my face, choking on my own breath. Arms pull me in and suddenly there is a barrier around me and my face is in his shoulder and just that is enough to create a refuge where I remember to exhale, and I am crying for real now, loud and gasping and uncontrollable. I am trembling and fluttering like an untethered sail and this is the raft to which I am clinging while I am buffeted by the aftermath of the storm.

It was one minute or five minutes or five years. And apparently I had survived the torrent of emotions because I slowly uncurled my fingers from the back of his scrub shirt and stepped back. I took another long drag from the cigarette. I turned away, embarrassed, because now I have to pick up my broken pieces and try to pretend I’m whole again. Because this is what we do. If I can’t handle this, I have no business being here.

“This is so dumb.” It’s the only thing I can manage to say, as I press the butt of the cigarette into the top of the metal railing. I feel my ears burning.

He punches me in the arm “Don’t be a dumbass. You’re too judgemental with yourself. Stop.”

I finally feel like I can get a full breath. I take a deep one in, and slowly release, as he asks again, quietly, “What happened?”

I shrug. I open my mouth and I intend to give some non-answer but then it just tumbles out - that it was nothing and everything and for them it was just a drill but how oblivious do you have to be to design a scenario that had us literally relive some of the worst parts of our professional lives. And that I am ashamed that this is the thing that got to me because it’s not like I haven’t seen shit and I haven’t seen people die horrible deaths and we’ve been through multiple fucking wars and this, this is the thing that set me off. Because my dumb amygdala doesn’t know that each experience is different, because it’s not it’s all one big stress response and despite all the therapy and effort I never have enough time or emotional capital to fortify and maintain the foundation that protects me from this loop, so I just have to anticipate its collapse at inopportune moments.

Again, since I’m remembering how, I exhale.

“Seems like your therapist is going to be getting some extra money for the vacation fund, eh?”

I crinkle my nose and chuckle despite myself. “If I ever find time to go see her.”

I look at him, and, inexplicably, I feel like the air I’m breathing is clearer, the shame I’m carrying is lighter. Because he gets it and he’s not judging me and he’s not pitying me and he’s not doing anything at all except that he didn’t leave and that is everything. Because I found a small patch of stable ground, a respite from the free fall, and I don’t have to stand here alone.

And for the rest of the day when everyone asks, I answer that I am fine because I am fine. I’m fine and this is what we do: we run our resilience Ponzi schemes and we pretend it’s not disintegrating beneath us, even as we renovate the facade with new paint and flowers and podcasts about burnout. Because even as I am falling, falling, falling off that cliff, once in a while I manage to grab on to a crack in the stone and drag myself, beaten and bloody, onto the ledge, where I see others like me and we punch each other in the arm and make bad jokes and rage and cry and share a cigarette and tell each other we’re going to be fine.

But we are not okay.

